# Adding Fuel to the Collective Fire: Stereotype Threat, Solidarity, and Support for Change

**DOI:** 10.1177/01461672231202630

**Published:** 2023-10-21

**Authors:** Clarissa I. Cortland, Zoe Kinias

**Affiliations:** 1 UCL School of Management, London, UK; 2 Ivey Business School, University of Western Ontario, London, Canada

**Keywords:** stereotype threat, women, support for organizational gender balance, ingroup solidarity, collective action

## Abstract

We hypothesize a yet-unstudied effect of experiencing systemic stereotype threat on women’s collective action efforts: igniting women’s support for other women and motivation to improve organizational gender balance. Hypotheses are supported in two surveys (Study 1: *N* = 1,365 business school alumnae; Study 2: *N* = 386 women Master of Business Administration [MBA]), and four experiments (Studies 3–6; total *N* = 1,897 working women). Studies 1 and 2 demonstrate that experiencing stereotype threat is negatively associated with women’s domain-relevant engagement (supporting extant work on the negative effects of stereotype threat), but positively associated with women’s support and advocacy of gender balance. Studies 3 to 6 provide causal evidence that stereotype threat activation leads to greater attitudes and intentions to support gender balance, ruling out negative affect as an alternative explanation and identifying ingroup solidarity as a mechanism. We discuss implications for working women, women leaders, and organizations striving to empower their entire workforce through developing equitable and inclusive practices.

Although gender balance in organizations has improved somewhat over the past decades (Badura et al., 2018; Graf et al., 2019), the persistence of gender gaps in economic opportunities and of women’s underrepresentation in top leadership positions (Catalyst, 2022) serve as stark reminders of the work remaining before worldwide economic gender equity can be achieved—a goal set to take 151 more years, according to the leading estimate (World Economic Forum [WEF], 2022). Despite best intentions, too often organizational efforts fall short in garnering the necessary momentum to sustain effective gender balance initiatives. We define gender balance as (a) equivalent numeric representation of women and men in all levels of any organizational system, (b) equal experiences of inclusion and belonging within organizational roles for women and men, and (c) equitable opportunities and rewards for women and men.

There is an assumption that women’s increasing presence within organizations and in leadership positions will bolster efforts to support gender balance and improve workplace experiences for other women. However, increasing the representation of women within a context does not unequivocally lead to beneficial outcomes for women (e.g., Ely, 1994; Joshi et al., 2015; Manzi & Heilman, 2021). In fact, there are many reasons why women might not universally support efforts to develop gender balance (e.g., to avoid interpersonal penalties for being perceived as less competent: Hekman et al., 2017; as self-interested: Gardner & Ryan, 2020; or as engaging in superfluous ingroup favoritism: Sidanius et al., 1994), or might not support other women (e.g., to avoid social comparison threat, Duguid et al., 2012).

To enhance gender balance initiatives’ success, understanding how and why women become motivated to support such initiatives and to support the professional advancement of other women at work is essential. We focus here on an important but neglected phenomenon that has previously been theorized and shown to have a *negative* impact on the motivation and engagement of women in the workplace: stereotype threat (ST). Decades of ST research have shown that women in male-dominated or male-typed contexts, and most notably in Science, Technology, Engineering, and Mathematics (STEM) as well as business and leadership domains, can experience *heightened concerns about being judged based on negative gender stereotypes*. These concerns can diminish women’s engagement and performance in negatively stereotyped domains (Brands & Fernandez-Mateo, 2017; Hoyt & Murphy, 2016; Shapiro & Williams, 2012; Spencer et al., 1999).

While research to date has largely focused on the unambiguously negative outcomes associated with ST, this literature has overlooked the possibility that ST may, in addition, trigger a *constructive* consequence for women: by igniting women’s motivations to support other women and take actions toward building gender balance. We develop the idea that ST can be a crucial motivator of women’s support of other women—or ingroup support—and their desire to contribute meaningfully to organizational change that advances gender balance. Having personally experienced psychological threat due to negative gender stereotypes may be fundamental in motivating women’s efforts to break down the professional barriers women face—especially with the hope of sparing other women from a similar fate.

We explicitly distinguish the experience of gender-based ST—heightened concerns about being judged based on negative gender stereotypes—from the related but theoretically distinct constructs of perceived (i.e., being aware of) gender bias and experienced gender bias. This distinction is important because whereas perceived and experienced bias have been well-established as predictors of collective action in the literature (Thomas et al., 2020; van Zomeren et al., 2008), ST has not. Failing to differentiate between ST and awareness of bias may undermine efforts to most effectively buffer the well-being of women while motivating change.

ST and bias awareness might be viewed as two sides of the same coin when it comes to women’s negative experiences in male-dominated contexts, both of which can coexist at individual and contextual levels. However, they may not always co-occur, as they rely on different triggers and cognitive processes. Becoming aware of gender bias due to observing or personally experiencing it enables the possibility of acknowledging the reality of prejudice (i.e., perceiving that women are underrepresented in a context or receiving negative evaluations generates attributions that others are biased), whereas ST concerns rely on appraisals of the self as a member of a negatively stereotyped group (Spencer et al., 2016). Because of this, one need not necessarily perceive bias or experience discrimination to feel threatened by a stereotype, suggesting that ST could motivate support for change even in the absence of acknowledged bias. Indeed, given recognizable barriers to perceiving and acknowledging bias in the first place—including ambiguity around attributing negative events to prejudice and retaliation concerns (Crocker et al., 1991; Swim & Hyers, 1999)—relying on bias-focused constructs to motivate change is limiting and neglects other important avenues for enacting change. We view the experience of ST as having a unique and important link to ingroup solidarity and support that is distinct from the consequences of bias awareness.

Our work offers three main insights into the rich and expansive literatures on ST, gender and social identity, and diversity in organizations. First, we show evidence of a constructive downstream consequence—ingroup support and collective action motivation—of ST, a phenomenon that has been previously regarded as having an overwhelmingly negative impact on cognitive and performance outcomes. The theoretical and practical implications of clarifying the range of outcomes resulting from ST are vast, particularly for diversity science researchers partnering with organizational leaders to build and implement transformational workplace interventions (Spencer et al., 2016). Second, we answer a call for research into how organizations can maximize support for gender balance in work settings (Gardner & Ryan, 2020). Finally, we offer a novel suggestion for how organizations can improve their diversity efforts. While the end goal for workplace Diversity, Equity, and Inclusion (DEI) initiatives should be to reduce and ultimately eliminate the possibility of experiencing ST, the present set of studies offers an interim recommendation for workplaces still struggling with DEI efforts. We suggest that giving voice to women’s ST narratives could be vital to fueling the fire of their collective action efforts.

## Gender Stereotypes Threaten Women’s Potential for Success

As women’s identities are threatened in contexts where women are underrepresented and negatively stereotyped, these systems can activate both acute and chronic concerns about confirming negative gender stereotypes in male-dominated domains such as mathematics (Shapiro et al., 2013), finance (von Hippel et al., 2015), leadership (Hoyt & Murphy, 2016), entrepreneurship (Gupta & Bhawe, 2007), and business broadly defined (Cortland & Kinias, 2019; Roberson & Kulik, 2007). Awareness of negative stereotypes that devalue women can trigger several interrelated psychological and cognitive processes for women—all of which have been associated with the experience of ST. The more acute form of ST triggers in-the-moment responses that include ruminating on negative thoughts, disrupted prefrontal processing, increased monitoring, thought suppression, and increased feelings of self-doubt—all resulting in the depletion of valuable cognitive processes required to perform well on challenging tasks (Hall et al., 2019; Kinias & Sim, 2016; Schmader et al., 2008).

Most extant research on ST has focused on measuring short-term task performance on SAT- or GRE-like standardized test questions in experimental laboratory settings where acute experiences of ST were intentionally activated (Nguyen & Ryan, 2008). Some applied work has also demonstrated evidence consistent with the impact of acute experiences of ST in real-world and/or professional settings (Block et al., 2011). For example, experimentally inducing conditions of ST led to women’s underperformance on a managerial task compared with men (Bergeron et al., 2006), as well as women business school students’ and entrepreneurs’ underperformance during a negotiation activity compared with men (Kray et al., 2001). Furthermore, a more chronic experience of ST may explain the objective performance decrements observed in contexts where women typically are underrepresented and negatively stereotyped, including professional education settings (e.g., business schools; Kim et al., 2022; Kinias & Sim, 2016) and actual workplaces (Joshi et al., 2015).

In addition to performance decrements, ST can also lead to women’s disengagement in professional domains. This includes psychological distancing through reduced aspirations and intentions to succeed in stereotyped domains (Davies et al., 2005; Gupta & Bhawe, 2007) and physical distancing by avoiding or exiting stereotyped domains including leadership, finance, consulting, and entrepreneurship (e.g., Barbulescu & Bidwell, 2013; Davies et al., 2005; Gupta & Bhawe, 2007). Furthermore, chronic and prolonged ST experienced over time predicted women’s intentions to quit in the legal profession (von Hippel et al., 2011) and women’s reduced recommendations for other women to join their own stereotypically masculine fields of finance and accounting (von Hippel et al., 2015).

Most previous work has focused on the effects of *either* acute experiences of ST *or* more chronic forms, but both forms are included in the broader concept of *systemic* ST (Block et al., 2019). This occurs “when an individual is in a system that is characterized by . . . gender disparities and the implicit belief about the reason for these disparities is due to stereotypes about deficits of individual group members rather than systemic inequality” (p. 35). The focus of the current work is to better understand the distal outcomes associated with this systemic form of ST, rather than to focus only on the proximal effect of a single, acute occurrence of ST. This is because we believe systemic ST more realistically portrays the lived experiences of ST, which do not happen in isolation but rather build on one another over time and lead to different patterns of performance, engagement, and coping strategies.

Taken together, the evidence thus far demonstrates that ST leads to negative professional outcomes—including domain-relevant disengagement—for women in stereotypically masculine work contexts such as competitive global business and leadership.

**Hypothesis 1 (H1):** ST negatively impacts women’s engagement in the negatively stereotyped domain.

What research on ST misses thus far, and what we contribute to the literature, is an exploration of a categorically different outcome for women navigating environments infused with ST. Specifically, we propose that there is a causal link between systemic ST and support for initiatives and efforts that drive gender balance.

## ST as a Predictor of Ingroup Solidarity and Support

In addition to the much-studied negative effects of ST on performance, motivation, and engagement, we propose that there is a crucial, not-yet-studied, and more constructive consequence of experiencing ST: its activation of women’s solidarity and support of other women, and of their collective efforts to enact social change to improve gender balance. Although no empirical work has demonstrated a causal link between ST and support for change, a significant body of work provides evidence consistent with the link between perceiving discrimination against one’s group and support for and engagement with collective action efforts to improve group conditions (e.g., social identity model of collective action, or SIMCA; Thomas et al., 2020; van Zomeren et al., 2008). For example, recognizing discrimination against their own group leads women and racial minorities to express positive intergroup attitudes toward other groups who are similarly discriminated against (Cortland et al., 2017). In addition, Derks and colleagues (2016) suggest that women’s awareness of gender discrimination at both the personal and group level should predict their support of other women, but acknowledge that women’s perceptions of gender discrimination generally tend to be low due to a variety of factors, including a lower likelihood of noticing when gender inequality personally impacts their own lives (Taylor et al., 1990). This suggests the importance of perceiving and recognizing the negative impact of gender bias at not only the aggregate level (i.e., perceiving group-level gender bias) but also at a personal level (i.e., personally experiencing gender bias).

Building beyond the extant literature, theoretically, the implications of systemic ST should be broader and more far-reaching, as individuals do not necessarily need to experience bias, or even perceive bias in their context, to feel threatened by a negative stereotype. ST is a cognitive and psychophysiological phenomenon that resides in the mind and body (Schmader et al., 2008) and does not rely on the presence or perception of discrimination to be activated. As Steele (1997) described it clearly, ST’s pernicious consequences result from “a threat in the air,” and its systemic form is an aggregation of experiences with these concerns of confirming negative stereotypes accumulating over time (Block et al., 2019) that do not require recognizing or being the target of bias. Thus, focusing on systemic ST as a motivator of support for change beyond perceived and experienced bias is necessary to capture an important predictor of collective action not incorporated in existing models (e.g., SIMCA).

Finally, in-depth interviews conducted with accomplished women scientists uncovered a theme that emerged among women navigating stereotype-threatening systems: Some women shared how they engaged in ST-confronting strategies that included advocacy efforts and supporting/collaborating with other women (Block et al., 2019). Taken together, these findings suggest that women’s motivation to help and support other women and to improve gender balance could directly result from personally experiencing ST. Women who recognize and acknowledge how systemic ST has affected them are thus predicted to develop a profound appreciation of the need for change.

Although no empirical research to date has looked at the causal effect of ST on activating responses consistent with ingroup support or promoting diversity, some work has shown how potential triggers of ST (e.g., exposure to overt sexism) can lead to increases in women’s endorsement of gender equality initiatives (Becker & Wright, 2011). Just as exposure to overt sexism forced women to acknowledge unambiguous prejudice against women and strive to combat it, acknowledging “the threat in the air” in the form of personal experiences with ST should lead to increased motivation to improve women’s plight.

**Hypothesis 2 (H2):** ST positively impacts women’s support of gender balance.

In considering process, we draw from two major theories grounded in intergroup relations research. First, social identity theory (SIT; Tajfel & Turner, 1986) posits that the most frequent self-esteem-restoring response to perceiving threats to one’s valued social identity is engaging in ingroup favoritism. Therefore, experiencing ST should motivate a desire to support and redeem one’s ingroup by committing to improving the status of the ingroup. SIT further proposes that there are three socio-structural variables that affect how people manage identity concerns and navigate identity threats: the permeability of group boundaries, the legitimacy of intergroup relations and status hierarchy, and the stability of the status hierarchy. To the extent that one’s disadvantaged group status is perceived as impermeable or immoveable, illegitimate, and unstable, this should lead to increased ingroup identification and increased motivation to engage in collective action to improve the ingroup’s status. Taken together, the propositions of SIT suggest that experiencing and perceiving the negative impact of ST—or the fear of being judged on the basis of gender-relevant stereotypes—should increase women’s feelings of ingroup solidarity and support.

SIT integrates with the SIMCA, the second theoretical model from which we draw to support the hypothesized process through which ST should lead to support for change. According to SIMCA (see also the collective action model of social change; Dixon et al., 2010; Wright & Lubensky, 2009), people are more likely to engage in collective action aimed at improving their group’s standing when they are highly committed to their group (high group identification), when they perceive their group faces injustice compared with other groups (high perceived injustice), and when they believe they can effectively enact change (high efficacy; van Zomeren et al., 2008). The resultant coalitionary motivation for members of disadvantaged groups to fight collectively for their group rights is termed “collective action orientation” (Wright & Lubensky, 2009) and hinges on the emergence of a common cause, or sense of solidarity (McGarty et al., 2009). Therefore, we propose that ST motivates collective action orientation just as perceiving injustice to one’s disadvantaged group does (e.g., Barlow et al., 2012) such that:

**Hypothesis 3 (H3):** The effect of ST on women’s support of gender balance is mediated by feelings of solidarity and common fate with other women.

In addition to ST leading to women’s disengagement in the negatively stereotyped domain, we propose a novel consequence of ST that has yet to be empirically tested: that ST increases women’s support of other women and support for increasing the advancement of women in leadership positions. Furthermore, we hypothesize that this motivation for change is explained by women’s collective solidarity, or perceived common fate, with other women. We expect that women’s drive to improve gender balance will arise from an impulse to spare other women from having to experience and be impacted by ST the same way they were.

Hypotheses were tested in six studies: two correlational survey studies examining women’s support for gender balance predicted by measured ST, and four experiments examining the extent to which manipulating ST causally increased women’s support for gender balance. In all six studies, we explored the consequences of ST for women in work contexts, focusing on ST as a driver of motivation and actions to develop gender balance.

## Study 1

Study 1 investigated Hypotheses 1 and 2 in a global sample of women with postgraduate business education. Women representing the full span of professional age generations reported gender-relevant ST experienced throughout their careers within diverse global locations and industries, thus testing our hypotheses in a sample that represented experiences accumulated across time and across heterogeneous organizational environments. We predicted that ST would be associated with reduced domain engagement—operationalized here as work satisfaction—and greater interest and involvement in efforts to advance gender balance.

### Method

We report all materials, manipulations, measures,^1^ and exclusions in all studies. Study 6 is the only study in this article that was preregistered. Data files, code books, and analysis codes for all studies are available on the Open Science Framework: https://osf.io/ts4xy/?view_only=1ff09c966f5441f79882b3a86244d00d

#### Participants and Procedure

Female alumnae (*N* = 1,365; modal age range: 42–57) of a graduate business school with campuses in Europe and Asia participated in an online survey. Alumnae respondents were globally diverse, representing 78 countries (no more than 15% of the sample from any one country), and with the majority holding regional or global responsibilities (69%) at professionally senior levels (70% worked in Chief Executive Officer [CEO]/C-Suite roles, as general managers, or reported directly to general management).

### Materials and Measures

#### ST

Participants indicated the extent to which they agreed with two items measuring gender-relevant ST on a scale from 1 = *strongly disagree* to 5 = *strongly agree* (adapted from Shapiro, 2011): (1) “At the most challenging stage of my career, I was concerned about confirming stereotypes about my gender”; and (2) “Currently, I am concerned about confirming stereotypes about my gender.” The combination of the two items aimed to assess experiences of ST over time and were averaged to create a composite measure (Pearson’s *r* = .55).

#### Domain Engagement

Participants reported their work satisfaction as an indicator of engagement in their work domain by responding to the following three items (Ely et al., 2014) on a scale from 1 = *not at all satisfied* to 5 = *extremely satisfied*: “At this stage in your life, how satisfied are you with the following: (1) Work that is meaningful; (2) Opportunities for career growth and development; and (3) Professional accomplishments.” Responses were averaged to create a work satisfaction score (α = .82).

Given that work engagement can arguably exist and be maintained outside of satisfaction with these three aspects of work, we collected an additional sample of women (from Prolific; *N* = 142) for the purposes of construct and measurement validation. We ran a confirmatory factor analysis (CFA) to assess the extent to which the current three items loaded onto a latent measure of domain engagement (referring primarily to work satisfaction) that correlated meaningfully with two alternative pre-established measures of domain engagement: (a) the Devaluing subscale of the Psychological Disengagement Scale (Major & Schmader, 1998) measuring the extent to which participants agreed it was important or unimportant to do well at work (five items, e.g., “Doing well at work is very important to me”; α = .87); and (b) the Career-Oriented Commitment scale (Ellemers et al., 1998) measuring the extent to which participants agreed they felt committed to the goal of advancing at work (four items, e.g., “My work plays a central role in my life”; α = .78). Results demonstrated that the three-item measure of work satisfaction used in this study correlated meaningfully (estimates >.5) with the two alternative measures of work engagement—as would be expected if the work satisfaction scale we used to measure domain engagement taps work engagement similarly to these established scales (see supplementary SOM file for full details). Thus, the items used here represent a valid proxy for domain (i.e., work) engagement.

#### Attitudinal Support for Gender Balance

Participants answered the following single questionnaire item on a scale from 1 = *I am not at all interested in these efforts* to 5 = *I am extremely interested in these efforts*: “How interested are you in efforts to increase the representation of women in high impact leadership positions?” We examined the validity of this single-item measure of attitudinal support by running a CFA with the same external sample of 142 participants described above. Results revealed that the standardized loading of the single item with the overall latent measurement of attitudinal support for gender balance was *β* = .87, *p* < .001 (see SOM for full details), indicating the target item used in this study taps into the same underlying latent variable as other established items assessing attitudinal support for gender balance (Bargad & Hyde, 1991; e.g., “I want to work to improve women’s status in business and society”) and serves as a valid short-form measure of attitudinal support.

#### Behavioral Support for Gender Balance

Participants indicated their behavioral contributions to improving gender balance in their organizations by responding on a scale from 1 = *no effort* to 5 = *an exceptional effort* to (1) “How would you describe the effort you invest, if any, in mentoring women?” (2) “How would you describe the effort you invest, if any, in advocating on behalf of women?” Responses were averaged to create a behavioral support score (*r* = .57).

### Results

Table 1 contains the descriptive statistics and correlations for all study variables. We conducted Ordinary Least Squares (OLS) regression analyses to investigate whether women’s concerns about confirming gender stereotypes during their careers significantly predicted domain disengagement and support for gender balance. Across studies, analyses were run both with and without covariates in the models and reported with covariates included^2^ (see Table 1 for descriptions of covariates).

**Table 1.** Study 1: Means, SDs, and Bivariate Correlations of Study Variables.

	*M*	*SD*	1	2	3	4	5	6	7	8
*N* = 1,365										
1. ST	2.93	1.172								
2. Work satisfaction	3.54	0.951	−.065*							
3. Attitudinal support	3.95	1.071	.230***	.093***						
4. Behavioral support	3.48	0.851	.169***	.168***	.391***					
5. Age (cov.)	1.74	0.700	−.135***	.069*	−.083**	.075*				
6. Job status (cov.)	4.36	1.899	−.014	.268***	.058*	.160***	.219***			
7. Degree program (cov.)	0.73	0.445	.076**	−.039	−.043	−.084**	−.234***	−.152***		
8. Company size (cov.)	8.23	3.918	.025	−.108***	.043	−.062*	−.222***	−.288***	−.020	
9. Job scope (cov.)	3.08	0.984	.033	.045	.097***	.099***	−.053	.052	−.012	−.017

*Note.* Covariates coded as follows: Age (Millennial = 1; Generation X = 2; Baby Boomer = 3; Older generations = 4); job status (CEO, president, or similar = 7; other C-Suite or similar = 6; other general management responsibilities = 5; report to general management = 4; mid-level manager = 3; team leader/project manager = 2; individual contributor = 1); degree program (non-MBA/executive degree program = 0; MBA = 1); company size (scale from 1–12: <5 employees = 1; 100–249 employees = 6; 10,000 or more employees = 12); and job scope (local = 1; national = 2; regional = 3; global = 4). SD = standard deviation; ST = stereotype threat

* *p* < .05. **p < .01. ***p < .001.

Results of hypothesis tests are displayed in Table 2. To test H1, workplace satisfaction was regressed on ST concerns. Consistent with past work, ST predicted women’s decreased workplace satisfaction, *b* = −.050, *SE* = .022, *t*(1,330) = −2.322, *p* = .020, 95% CI [−.092, −.008].

**Table 2.** Study 1: Estimates From Series of Linear Regressions.

Predictors	Work satisfaction			Attitudinal support			Behavioral support		
	*β*	*b*	*SE*	*β*	*b*	*SE*	*β*	*b*	*SE*
Intercept	—	3.63**	.05	—	3.95**	.05	—	3.54**	.05
Age	−.01	−.01	.04	−.07*	−.11*	.04	.04	.05	.04
Job status	.26***	.13***	.01	.08**	.04**	.02	.14***	.06***	.01
Degree program	.01	.01	.06	−.07**	−.18**	.07	−.07*	−.14*	.06
Company size	−.03	−.01	.01	.04	.01	.01	−.01	−.00	.01
Job scope	.03	.03	.03	.08**	.08**	.03	.08**	.07**	.03
ST	−.06*	−.05*	.02	.22***	.20***	.02	.18***	.13***	.02
*R* ^2^	.08			.08			.07		
*F* for change in *R*^2^	18.98***			18.35***			13.99***		

*Note.* All predictors were centered according to the overall sample means. The *β* estimates refer to the standardized regression coefficients.

The *B* estimates refer to unstandardized regression coefficients. *SE* = standard error; ST = stereotype threat.

* *p* < .05. **p < .01. ***p < .001.

Next, we tested H2 by regressing both attitudinal and behavioral support for gender balance on women’s ST concerns. Supporting H2, ST concerns predicted increased attitudinal support of gender balance, *b* = .205, *SE* = .024, *t*(1,337) = 8.474, *p* < .001, 95% CI [.157, .252]. Women’s increased concerns about confirming gender stereotypes in their careers also predicted their efforts to promote gender balance by mentoring and advocating for other women, *b* = .133, *SE* = .021, *t*(1,098) = 6.217, *p* < .001, 95% CI [.091, .175].

### Discussion

Consistent with H1 and prior work on gender and ST in organizational contexts, ST concerns predicted reduced work satisfaction in a sample of established professional women, the majority of whom were managers and leaders in their businesses. Moreover, supporting H2 and providing the first evidence consistent with a constructive consequence of ST, women who experienced heightened ST concerns were more supportive of gender balance.

Study 1 established the relationship between ST and support for gender balance across heterogeneous work contexts in terms of country, industry, company size, and women’s seniority level. One limitation of Study 1, however, is that the heterogeneity represented could have introduced unmeasured confounding factors. The goal of Study 2 was to address this limitation.

## Study 2

Study 2 aimed to replicate and extend the core findings of Study 1 in a sample of women MBA students, focusing on their immediate school context. We predicted that women MBA who reported more ST at their school would report reduced domain engagement—operationalized as feelings of commitment to their school and willingness to recommend their school to prospective female MBA students (H1). We further hypothesized that ST would predict women’s increased interest and involvement in efforts to improve the gender climate at their school (H2). Moreover, we measured and controlled for two related constructs: perceived gender bias and experienced gender bias. Including these measures allowed for statistical isolation of the unique effect of ST after parsing out any effects that perceiving and experiencing gender bias may have on the focal outcomes.

### Method

#### Participants and Procedure

Female MBA students (*N* = 386) at the same business school as Study 1 participated anonymously in an online survey across 3 years of data collection (2017–2019). The survey was administered to better understand students’ perceptions of the gender climate in their current MBA program.^3^

### Materials and Measures

#### ST

Participants indicated the extent to which they agreed with the following on a scale from 1 = *strongly disagree* to 5 = *strongly agree* (adapted from Shapiro, 2011): (1) “I sometimes worry that people at [*School*] feel that I have less ability because of my gender”; and (2) “At [*School*], sometimes I am concerned about confirming stereotypes about my gender group.” Analyzing these items separately did not result in any meaningful changes to the pattern of findings, so responses were averaged to create a composite measure of ST (*r* = .60).

#### Perceived and Experienced Gender Bias

Participants indicated perceptions of gender bias in their school by responding to the question: “How gender biased in general is [*School*]?” on a scale from 1 = *biased in favor of women* to 3 = *no bias* to 5 = *biased in favor of men.* Participants then indicated personal experiences of gender bias by responding *no* = 0 or *yes* = 1 to “Have you ever personally experienced gender bias at [*School*]?” We ran CFAs to validate these single-item measures of perceived and experienced gender bias against established three-item measures of perceived gender bias (adapted from Thomas et al., 2020; e.g., “Do you think people from your gender group are discriminated against at your work?”; α = .93) and experienced gender bias (adapted from Thomas et al., 2020; e.g., “Have you personally been discriminated against due to your gender at work?”; α = .94) in a separate sample of women recruited via Prolific (*N* = 142). Results revealed significant standardized loadings of the single items with the overall latent measurements of perceived bias (*β* = .64, *p* < .001) and experienced bias items (*β* = .68, *p* < .001; see SOM file for details), consistent with the interpretation that the single-item measures used in this study represent valid short-form measures of perceived and experienced gender bias.

#### Domain Engagement

We measured participants’ school engagement via two variables, analyzed and reported separately: school commitment and willingness to recommend their school to prospective female MBA students. Five items measured school commitment (adapted from the Affective Commitment subscale of the Organizational Commitment Scale; Allen & Meyer, 1990) on a scale from 1 = *strongly disagree* to 5 = *strongly agree*: (1) “I am glad I chose to come to [*School*]”; (2) “I have a strong sense of belonging at [*School*]”; (3) “I add value at [*School*] and it suits my personal development needs”; (4) “I feel empowered to make important decisions at [*School*]”; and (5) “I feel included and respected at [*School*].” Responses were averaged to create a school commitment score (α = .86). Because school engagement might arguably exist outside of agreement with the particular items comprising school commitment measured here, we ran a CFA to assess the fit of a model in which the current five items loaded onto a latent construct of domain engagement (referring primarily to commitment) and correlated with the same two measures of domain engagement (valuing work and career-oriented commitment) used to validate work satisfaction in Study 1. Results revealed that the five-item measure of commitment used in this study correlated meaningfully (estimates >.5) with both alternate measures of domain engagement (see SOM for details), demonstrating the items used in this study tap domain (school) engagement similarly to other established measures of domain engagement.

Recommending their school to similar others is a measure of net promoter score, which is an indicator of loyalty—another component of commitment reflected in the Normative Commitment subscale of the Organizational Commitment Scale (Allen & Meyer, 1990). Furthermore, recommendations have been used in previous work as a key outcome variable indicating engagement and have been found to be negatively predicted by ST (e.g., von Hippel et al., 2015). Thus, in the current study, participants indicated on the same 5-point scale as above the extent to which they agreed with the statement: “I would recommend coming to [*School*] to women who are considering MBA programs.”^4^

#### Support for Gender Balance

All participants indicated their support for efforts to achieve gender balance by responding to the following question: “Would you like to see efforts to improve the gender climate at [*School*]?” Response options were 1 = *no*, 2 = *maybe*, and 3 = *yes*. We examined the validity of this single-item measure of attitudinal support by running a CFA with an external sample of 142 participants. Results revealed that the standardized loading of the single item with the overall latent measure of attitudinal support for gender balance was *β* = .82, *p* < .001 (see SOM for details), indicating our single-item measure represents a valid short-form measure of attitudinal support for gender balance.

Participants in the second and third years of data collection also reported their own behavioral contributions to gender balance by answering on a scale from 1 = *not at all* to 5 = *a great deal*: “How much do you contribute to efforts to improve the gender climate at [*School*], either formally or informally?” A separate CFA conducted in an external sample of participants (*N* = 142 women) demonstrated this single item loaded significantly (along with the same two-item measure of behavioral support used in Study 1) onto the latent construct of behavioral support (*β* = .82, *p* < .001), demonstrating its validity as a short-form proxy measure for the construct of behavioral support for gender balance (see SOM for details).

### Results

Table 3 contains the descriptive statistics and correlations for all study variables. A series of OLS regression analyses investigated H1 and H2. To isolate the predictive power of ST and control for conceptually related constructs of perceiving and experiencing gender bias, as well as potential time, cohort, and campus effects, several covariates were included (note that excluding covariates did not change any of the patterns or statistical significance levels; see SOM). Providing support for H1, women’s ST concerns significantly and negatively predicted their school commitment, *b* = −.085, *SE* = .037, *t*(378) = −2.278, *p* = .023, 95% CI [−.159, −.012]. Moreover, women’s ST concerns significantly predicted their reduced willingness to recommend their school to prospective female MBA students, *b* = −.166, *SE* = .045, *t*(378) = −3.713, *p* < .001, 95% CI [−.254, −.078].

**Table 3.** Study 2: Means, SDs, and Bivariate Correlations of Study Variables.

	*M*	*SD*	1	2	3	4	5	6	7	8	9	10
*N* = 386												
1. ST	3.00	1.133										
2. Perceived bias	3.47	0.850	.331***									
3. Experienced bias	0.53	0.499	.411***	.394***								
4. School commitment	3.94	0.791	−.178***	−.309***	−.136**							
5. Recomm. to women	4.35	0.934	−.237***	−.237***	−.190***	.603***						
6. Attitudinal support	2.58	0.642	.338***	.459***	.342***	−.118*	−.131**					
7. Behavioral support	2.92	1.047	.271***	.289***	.361***	−.126	−.145*	.306***				
8. Year 2017 dummy (cov)	0.47	0.499	−.196***	−.011	−.115*	−.138**	−.144**	−.194***	—			
9. Year 2018 dummy (cov)	0.27	0.446	.049	−.200***	−.026	.185***	.114*	−.028	−.322***	−.573***		
10. Cohort (cov)	0.56	0.498	−.108*	−.194***	−.146**	−.124*	−.053	−.160**	−.031	.053	−.063	
11. Campus (cov)	0.53	0.500	.109*	.080	.125*	−.005	−.002	.117*	.125	−.178***	.105*	−.191***

*Note.* A new cohort of students began the 10-month MBA program in August and another one in January every year, so that at the time of data collection for each of the 3 years, two different cohorts participated in the survey: one cohort was in its eighth or ninth month (coded “1”) and the other in its third or fourth month (coded “0”). Campus was coded as 0 = Asia and 1 = Europe. The measure of “Behavioral Support” was collected only in the second and third years of data collection. SD = standard deviation; ST = stereotype threat.

* *p* < .05. **p < .01. ***p < .001.

Supporting H2, ST significantly predicted women’s interest in efforts to improve their school’s gender climate, *b* = .088, *SE* = .028, *t*(374) = 3.173, *p* = .002, 95% CI [.033, .142], and their self-reported behavioral contribution to improving their school’s gender climate, *b* = .130, *SE* = .063, *t*(205) = 2.072, *p* = .040, 95% CI [.006, .254] (see Table 4 for regression results).

**Table 4.** Study 2: Estimates From Series of Linear Regressions.

Predictors	School commitment			Recommend to women			Attitudinal support			Behavioral support		
	*β*	*b*	*SE*	*β*	*b*	*SE*	*β*	*b*	*SE*	*β*	*b*	*SE*
Intercept	—	4.31***	.12	—	4.81***	.14	—	2.58***	.09	—	2.69***	.17
Year 2017	−.14*	−.22*	.10	−.21**	−.39**	.11	−.19**	−.24**	.07	—	—	—
Year 2018	.04	.08	.11	−.04	−.07	.13	−.08	−.12	.08	−.25***	−.53***	.13
Cohort	−.19***	−.30***	.08	−.11*	−.20	.09	−.06	−.08	.06	−.00	−.00	.13
Starting campus	−.03	−.05	.08	−.00	−.00	.09	.01	.01	.06	.09	.19	.13
Experienced bias	−.01	−.01	.09	−.09	−.16	.10	.12*	.15*	.06	.24***	.52***	.14
Perceived bias	−.29***	−.27***	.05	−.15**	−.17**	.06	.33***	.25***	.04	.09	.10	.08
ST	−.12*	−.09*	.04	−.20***	−.17***	.05	.16**	.09**	.03	.14*	.13*	.06
*R* ^2^	.16			.13			.30			.25		
*F* for change in *R*^2^	5.19*			13.79***			10.07**			4.29*		

*Note.* All continuous predictors were centered according to the overall sample means. The *β* estimates refer to the standardized regression coefficients. The *B* estimates refer to unstandardized regression coefficients. *SE* = standard error; ST = stereotype threat.

* *p* < .05,.**p < .01. ***p < .001.

### Discussion

Study 2 conceptually replicated findings from Study 1 while controlling for related constructs of perceiving and experiencing gender bias. Consistent with the disengagement hypothesis of ST (H1) and prior work (e.g., von Hippel et al., 2015), women MBA’s concerns about confirming gender stereotypes predicted negative outcomes in terms of less school commitment and lower likelihood of recommending their school to other women. Supporting H2, heightened ST concerns predicted women’s increased interest in and self-reported contribution to improving the gender climate at their school.

Recall that these findings signal the unique effect of ST on support for gender balance after controlling for the effects of perceiving and experiencing gender bias at the school. Although both perceiving bias and experiencing bias appear to have their own unique effects on support for gender balance (see Table 4), ST itself explained a significant amount of variance in desire for and action toward improving gender balance, over and above these constructs. This is particularly notable because, theoretically, the implications of ST should be broader and more far-reaching than perceiving or experiencing gender bias, as individuals do not necessarily need to perceive bias in their context, or experience bias, to feel threatened by a stereotype.

In sum, Studies 1 and 2 uncover the as-yet-unstudied relationship between ST and women’s support for diversity among current and aspiring women business leaders. However, the correlational designs of Studies 1 and 2 limit causal interpretations. Therefore, the following four experiments manipulated the salience of ST experiences and measured the causal effects on women’s support for gender balance.

## Study 3

Study 3 investigated the impact of experimentally manipulated activation of ST concerns on women’s increased drive to improve gender balance among convenience samples of working women in the United States. This enables a causal test, also examining generalizability beyond business school students and alumnae.

### Method

#### Participants and Procedure

In all experiments (Studies 3–6), we aimed to recruit at least 150 participants per condition. An *a priori* power analysis using G*Power indicated a total sample size of at least 210 participants needed to have 95% power to detect medium effect sizes (*d* = 0.25) with alpha set at 0.05, but we planned to oversample to accommodate expected data exclusions. Working adult women in the United States (*N* = 540; mean age range: 30–39; 76% White, 7.5% African American/Black, 4% Hispanic/Latinx, 4% Asian/Asian American, 7.5% multiracial, all other <1%) were recruited from Prolific and MTurk to participate in a paid online study. Six participants were excluded for not completing the writing task, one for failing an attention check, 13 for not working at least part-time, and 14 for missing data, resulting in a final sample of *N* = 506. (See Table 4 in SOM for a summary of cell means and distribution of exclusions across conditions for Studies 3–6.)

Participants were randomly assigned to one of two conditions (ST, control) in a between-participants experimental design. Participants read that they would complete three unrelated tasks. The first task was the ST manipulation, framed as a written reflection exercise about personal and/or work experiences. Following Shapiro et al. (2013) procedures adapted from the academic to the work context, participants in the ST condition wrote about a time at work when they were being evaluated and were concerned about being judged on the basis of negative stereotypes about women (see SOM for exact instructions). Recalling and re-living a ST experience in this way was expected to produce similar psychological effects in real time that one might expect in an actual lived situation (Shapiro et al., 2013). Although most published ST experiments manipulate ST by reducing it since it exists “in the air” at baseline (Spencer et al., 2016), because we focus on a more chronic, systemic form of ST, our goal was to make the existing ST salient. In the control condition, participants wrote about what they did last Tuesday.

The second part of the study contained the dependent measures and was framed as sharing personal and work-related thoughts and opinions. Finally, participants answered demographics questions in the third part. After completing all three parts, participants read the debriefing and submitted responses.

### Materials and Measures

#### Attitudinal Support for Gender Balance

Similar to Study 2, participants indicated their interest in improving gender balance by answering, “Would you like to see efforts to increase the representation of women in high impact leadership positions in your current industry or area of work?” Responses were 1 = *no*, 2 = *maybe*, and 3 = *yes*. We preserved this item from Study 2 to test reliability across studies and generalizability of the findings among different sample populations. Recognizing the limitations of this single item with three response options, however, we include in Studies 5 and 6 an additional measure of attitudinal support that combines the average of two additional items and uses more traditional Likert-type scale response options.

#### Behavioral Intent to Support Gender Balance

Participants indicated their behavioral intentions to support gender balance on items adapted from Study 1, on a scale from 1 = *not at all interested* to 5 = *extremely interested*: “How interested are you in investing your own personal time and effort into (1) . . . mentoring and developing women and/or girls for a successful career in your profession?” (2) . . . advocating on behalf of women and/or girls for a successful career in your profession?” Responses were averaged together to create a behavioral intent to support score (*r* = .85).

### Results and Discussion

To test the hypothesis that ST affects women’s attitudinal and behavioral intent to support gender balance (H2), we conducted analyses of covariance (ANCOVA) investigating mean differences between conditions, statistically controlling for demographic characteristics (age, education, racial/ethnic minority status, and political orientation).^5^ Supporting H2, the ST manipulation increased women’s desire to increase the representation of women in leadership positions (*M* = 2.78, *SE* = .033) relative to the control condition (*M* = 2.66, *SE* = .031), *F*(1, 499) = 6.211, *p* = .013, *η_p_*^2^ = .012. Furthermore, the ST condition increased women’s interest in mentoring and advocating for women (*M* = 3.46, *SE* = .079) compared with the control condition (*M* = 3.20, *SE* = .073), *F*(1, 499) = 5.737, *p* = .017, *η_p_*^2^ = .011.

Study 3 causally demonstrated that activating ST increased motivation and advocacy to improve gender balance among working women. Compared with a no-threat condition, women for whom threat rooted in negative gender stereotypes was made salient reported increased motivation to correct gender inequality. However, one possible critique of Study 3 is that although the manipulation was designed specifically to situationally activate ST, it might also induce a negative mood, and this negative mood could motivate increased support of gender balance (e.g., anger and frustration as approach-related affect: Carver & Harmon-Jones, 2009). We argue rather than negative affect motivating women’s support, the specific psychological experience resulting from activating concerns about reinforcing gender stereotypes is what motivates women to support gender balance.

## Study 4

Study 4 tested H2 against the alternative explanation that negative affect activated women’s motivation to support gender balance using a similar experimental paradigm as Study 3 (same ST and neutral control conditions) plus a third condition where participants reflected on a negative work experience not explicitly related to gender. We predicted that feeling negative workplace affect would not influence women’s support of gender balance relative to a neutral control condition, whereas recalling a ST experience would increase women’s support for gender balance compared with the neutral control and negative affect conditions.

Furthermore, because ST might motivate women to help more broadly to behaviorally align with the gender stereotype associating women with communality (Akinola et al., 2018), in addition to measuring women’s intentions to mentor and sponsor other women, we also measured intentions to mentor and sponsor men. ST was predicted to motivate support only with respect to the negatively stereotyped ingroup—women.

### Method

#### Participants and Procedure

Working adult women in the United States (*N* = 664; mean age range: 30–39; 70% White, 9% African American/Black, 5% Hispanic/Latinx, 6.5% Asian/Asian American, 9% multiracial, all other <0.5%) were recruited through Prolific to participate in a paid online study. Twelve participants were excluded for not completing the writing task, 74 for not working at least part-time, and two for failing the attention check, resulting in a final sample of 576. Participants were randomly assigned to one of three conditions (ST, control, negative affect) in a between-participants experimental design. All study procedures and materials were identical to Study 3, with an additional third *negative affect condition*, wherein women wrote about a time when they failed to reach a goal at work (see SOM for exact instructions). Then, participants completed the dependent measures, including a measure of intentions to support men. Finally, participants answered demographics questions, read the debriefing, and submitted responses.

### Materials and Measures

#### Attitudinal Support for Gender Balance

Participants indicated their interest in improving gender balance by answering the same item as in Study 3.

An additional five-item measure of organizational policy support was included in this study (example item: “A ‘tie-breaker’ policy in which a woman is selected over a male applicant when the two applicants are equally qualified.”). Participants indicated their level of opposition or support to the five policy proposals on a scale ranging from 1 = *strongly oppose* to 7 = *strongly support*. However, the five policy items did not intercorrelate well enough together to form a reliable scale (α = .50), so we report the details of the individual items and analyses of each separate item as an outcome in the SOM.

#### Behavioral Intent to Support Gender Balance

Participants indicated their intentions to behaviorally contribute to improving gender balance in their profession by responding to the same two items as in Study 3, averaged together (*r* = .80).

#### Behavioral Intent to Support Men

Participants indicated their intentions to behaviorally support men in their profession by responding to two items similar to the behavioral intent to support gender balance items but replacing “women and/or girls” with “men and/or boys.” Responses were averaged to create a behavioral intent to support men score (*r* = .85).

### Results and Discussion

ANCOVAs were conducted to investigate the effect of ST on *attitudinal support* for gender balance relative to the neutral control and negative work affect conditions, statistically controlling for demographic factors. Although the omnibus test was not significant, *F*(2, 568) = 2.761, *p* = .064, *η_p_*^2^ = .010, the most critical *a priori* planned contrasts were. Women’s desire to increase the representation of women in leadership was higher in the ST condition (*M* = 2.77, *SE* = .042) relative to the control condition (*M* = 2.66, *SE* = .037), *F*(1, 568) = 3.874, *p* = .050, *η_p_*^2^ = .007, and relative to the negative affect condition (*M* = 2.64, *SE* = .039), *F*(1, 568) = 4.647, *p* = .032, *η_p_*^2^ = .008. The mean difference in attitudinal support of gender balance between the neutral control and negative affect conditions was not significant (*p* = .796). Finally, the ST condition increased women’s attitudinal support for gender balance in leadership relative to the other two conditions combined, *F*(1, 568) = 5.489, *p* = .019, *η_p_*^2^ = .010.

There were no consistent differences among and between the condition means for organizational policy support—analyzed as either a composite measure or looking at each of the five items individually (all *p* > .05; see SOM for details).

In the same set of analyses for *behavioral intent to support* gender balance, the omnibus test was again nonsignificant, *F*(2, 568) = 2.965, *p* = .052, *η_p_*^2^ = .010. Planned contrast analyses, however, revealed that the ST condition increased women’s interest in mentoring and advocating for women (*M* = 3.26, *SE* = .090) relative to the control condition (*M* = 2.97, *SE* = .079), *F*(1, 568) = 5.793, *p* = .016, *η_p_*^2^ = .010, and the negative affect condition (*M* = 3.06, *SE* = .083), though this latter difference was not statistically significant, *F*(1, 568) = 2.673, *p* = .103, *η_p_*^2^ = .005. As predicted, there was no difference between the control and the negative affect conditions (*p* = .451), and ST significantly increased intent to support women compared with the other two conditions combined, *F*(1, 568) = 5.238, *p* = .022, *η_p_*^2^ = .009.

Finally, we analyzed women’s *behavioral intent to support men and/or boys* in their industry/profession. The omnibus test and all four planned contrasts revealed no significant differences (all *p* > .4), demonstrating that the hypothesized effect pertains specifically to advancing women, and ruling out the alternative explanation that the ST manipulation merely induced women’s motivation to be supportive (of both men and women) to align with gender stereotypes.

Study 4 replicated and clarified experimental findings from Study 3, providing further causal evidence that ST increases motivation and advocacy to improve gender balance. Furthermore, the negative affect condition did not increase women’s support for gender balance, and no meaningful differences between the negative affect and neutral control conditions emerged on any of the focal dependent measures.

## Study 5

Studies 1 through 4 demonstrated that women’s experiences with ST increased their support for gender balance in business and leadership. Although ample work has investigated the explanatory processes involved in the deleterious effects of ST (Schmader et al., 2008), no empirical work has tested the impact of ST on women’s drive to impact social change. Study 5 aimed to test H3, that a sense of common fate (i.e., shared outcomes) and solidarity with ingroup others will activate a coalitionary mindset, leading to increased support for social change benefiting the collective ingroup.

### Method

#### Participants and Procedure

Working adult women in the United States (*N* = 449; mean age range: ages 30–39; 73.5% White, 9% African American/Black, 5% Hispanic/Latinx, 5% Asian/Asian American, 7% multiracial, all other <0.5%) were recruited through Prolific to participate in a paid online study. Eighteen participants were excluded for failing to complete the writing task, two for missing data on at least one of the analyzed variables, and 49 for not working at least part-time, resulting in a final sample of 380. Study procedures were the same as Study 3, with the inclusion of the mediating variable measuring common fate and an additional attitudinal measure of gender balance support.

### Materials and Measures

#### Common Fate

Participants responded to four items (adapted from published measures of common fate/common cause: Craig & Richeson, 2012; Subašić et al., 2018) on a scale from 1 = *not at all* to 7 = *extremely*: (1) “To what extent do you experience common outcomes and goals with other women?” (2) “To what extent is your personal success linked to the success of other women?” (3) “How much does your success depend on the success of women in general?” (4) “How much do you feel a sense of solidarity with other women?” Responses were averaged to create a composite measure of common fate (α = .83).

#### Attitudinal Support for Gender Balance

Participants indicated their interest in improving gender balance by answering the same item in Studies 3 and 4. To improve validity beyond this single item, participants responded to two additional items (adapted from Bargad & Hyde, 1991) on a scale from 1 = *strongly disagree* to 7 = *strongly agree*: (1) “I want to work to improve women’s status in business and society” and (2) “I care very deeply about men and women having equal opportunities in all respects.” Responses were averaged to create a second measure of attitudinal support (*r* = .55).

#### Behavioral Intent to Support Gender Balance

Participants responded to the same items used in Studies 3 and 4 (*r* = .79).

### Results and Discussion

First, ANCOVAs investigated the mean difference between conditions for all measures, controlling for demographics. Supporting H2, women’s interest in improving gender balance in high-impact leadership positions was higher in the ST condition (*M* = 2.75, *SE* = .038) versus the control condition (*M* = 2.62, *SE* = .039), *F*(1, 374) = 5.408, *p* = .021, *η_p_*^2^ = .014. Furthermore, women’s attitudinal support for gender equality measured via the additional two-item composite measure was also higher in the ST condition (*M* = 6.01, *SE* = .062) versus the control condition (*M* = 5.71, *SE* = .065), *F*(1, 374) = 11.554, *p* < .001, *η_p_*^2^ = .030. The ST condition increased women’s interest in mentoring and advocating for women (*M* = 3.18, *SE* = .081) relative to the control condition (*M* = 2.87, *SE* = .085), *F*(1, 374) = 6.656, *p* = .010, *η_p_*^2^ = .017. Finally, ST increased women’s perceived common fate with other women (*M* = 4.87, *SE* = .082) compared with the control condition (*M* = 4.60, *SE* = .086), *F*(1, 374) = 4.907, *p* = .027, *η_p_*^2^ = .013.

The PROCESS macro (Model 4; Hayes, 2012) tested the significance of the indirect pathway from ST to support for gender balance through the mediator of perceived common fate with other women (H3). Three models were specified testing the mediating process on each of three outcomes: (a) interest in increasing female representation in leadership positions; (b) attitudinal support for gender equality (additional two-item composite); and (c) behavioral intentions to mentor and advocate for women. Each PROCESS command was run with bootstrapping specified at 10,000 samples and demographics entered as covariates.

Results supported H3: Perceived common fate with other women significantly mediated the relationship between ST and support for gender balance—analyzed via two measures of attitudinal support and a third measure of behavioral intentions (see Table 5 for all path coefficients and confidence intervals). These findings suggest that perceiving solidarity with other women can explain why experiences with ST increase women’s social change motivations to achieve gender balance. Given established shortcomings of measurement-of-mediation designs, however, including the possible omission of confounding variables and susceptibility to alternative explanations, Study 6 seeks to adopt the experimental mediation approach of manipulating the hypothesized mediator (Pirlott & MacKinnon, 2016; Spencer et al., 2005).

**Table 5.** Study 5: Path Coefficients and Confidence Intervals of Mediational Models, *N* = 380.

	Attitudinal support for gender balance (single item)	Attitudinal support for gender balance (two-item composite)	Behavioral intent to support gender balance
*a*	.265* (.119)	.265* (.119)	.265* (.119)
*b*	.114*** (.023)	.380*** (.034)	.477*** (.045)
*c*	.127* (.055)	.307*** (.090)	.306* (.118)
*c’*	.097 (.053)	.206** (.079)	.179 (.104)
95% CI of the indirect effect	[.003, .066]	[.011, .192]	[.013, .244]

*Note. a* denotes the path of the effect of ST experience salience on perceived common fate with women. *b* denotes the path of the mediator’s (perceived common fate) effect on the dependent variable. *C’* denotes the direct effect of ST experience salience on the dependent variable. *c* denotes the total effect of ST experience salience on the dependent variable. Standard errors are in parentheses. CI = confidence interval.

* *p* < .05. **p < .01. ***p < .001.

## Study 6

Study 6 is a preregistered experiment conceptually replicating the mediational model tested in Study 5 in a sample of professional women managers/supervisors. To more directly test the proposed mediator of ingroup solidarity (i.e., common fate), and in conjunction with Study 5, we employ a double randomization, manipulation-of-mediator study design (see Spencer et al., 2005). Study preregistration can be found at https://osf.io/tuqfs/?view_only=da82a2f4e9aa4a958e8792088922c9f4.^6^

### Method

#### Participants and Procedure

Working adult women managers/supervisors in the United States (*N* = 494; mean age range: ages 30–39; 77.5% White, 10% African American/Black, 3% Hispanic/Latinx, 3% Asian/Asian American, 6% multiracial, all other <0.5%) were recruited through Prolific to participate in a paid online study. We focus here on women in leadership positions to provide a more direct test of hypotheses in a relevant and compelling sample. Therefore, only women who indicated in Prolific’s built-in prescreen that they have both management experience and roles involving leadership/power/supervisory duties were permitted to participate. Furthermore, 29 participants were excluded for self-reporting at the end of the study having no management experience at work. In addition, nine participants were excluded for failing to complete the writing task, three were excluded for failing the attention check survey question, and 18 were excluded for not working at least part-time, resulting in a final sample of *N* = 435.

Study procedures were similar to Studies 3 through 5, with some notable deviations. Participants were randomly assigned to one of three conditions (ST-Similarities, ST-Differences, and Control) in a between-participants experimental design. Participants in both ST conditions first completed the same ST manipulation used in Studies 3 through 5, framed as a written reflection exercise about personal and/or work experiences with ST. New to this study, after manipulating ST via the reflection task, participants were asked to reflect further on how the ST experiences they just wrote about are similar to (ST-Similarities) or different from (ST-Differences) the experiences of other women in similar work roles. This additional reflection task was meant to “turn on” or encourage the effect of the proposed mediator of common fate/ingroup solidarity in the ST-Similarities condition, and “turn off” or discourage the effect of the mediator in the ST-Differences condition. The ST-Similarities condition reflects an operationalization of the mediator at a high level—high common fate with women—and the ST-Differences condition reflects an operationalization of the mediator at a low level—low common fate. Thus, any mean gaps in support for gender balance as a function of conditions of the manipulated mediator would demonstrate causal support for the hypotheses. In the Control condition, similar to Studies 3 through 5, participants wrote about what they did last Tuesday, and, in addition, were asked to reflect on any similarities or differences between their Tuesday experiences and the typical Tuesdays of other people. Participants then completed the dependent measures, followed by the demographics questions and the debriefing, before submitting their responses.

### Materials and Measures

#### Attitudinal Support for Gender Balance

Participants indicated their interest in improving gender balance by answering the following four items used in Studies 1 through 5, all measured on a scale from 1 = *strongly disagree* to 7 = *strongly agree*: (1) “I would like to see efforts to increase the representation of women in high impact leadership positions in my current industry or area of work”; (2) “I would like to see efforts to improve the gender climate at my work”; (3) “I want to work to improve women’s status in business and society” and (4) “I care very deeply about men and women having equal opportunities in all respects.” Responses were averaged to create a composite measure of attitudinal support (α = .90).

#### Behavioral Intent to Support Gender Balance

Participants responded to the same two items used in Studies 3 through 5 (*r* = .84). To replicate and extend the effects of prior studies, two additional measures of behavioral intent to support gender balance were included: a measure of workplace policy support using a work vignette scenario and a measure of collective action tendencies directed at addressing the underlying causes of gender inequality (e.g., signing a petition and participating in a demonstration). For the measure of policy support, participants were asked to imagine a scenario in which their CEO has announced a goal to implement a new policy seeking to increase gender equity in the workplace, and the CEO was forming a task force to help determine any necessary policy changes (see Wang et al., 2021). Participants’ likelihood to undertake the following six actions in support of the policy were measured on a scale from 1 = *very unlikely* to 7 = *very likely*: (1) “Join the task force”; (2) “Try to recruit others to join the task force”; (3) “Volunteer your efforts to help the task force, even if it means extra work for you (on top of your normal work)”; (4) “Spend time researching diversity practices so that you can lend insight to the task force”; (5) “Post on social media in support of the aims of the task force”; and (6) “Vocalize your support in a meeting with all the staff of the organization.” Responses were averaged to create a composite measure of policy support (α = .93), with higher numbers indicating greater policy support.

Participants indicated their likelihood to engage in the following six future collective action behaviors in support of gender equality on a scale from 1 = *very unlikely* to 7 = *very likely*: (1) “Vote for political candidates who make gender inequality one of their serious concerns”; (2) “Sign a petition for more gender equality”; (3) “Participate in demonstrations that call for gender equality”; (4) “Join a group of activists demanding more gender equality”; (5) “Attend events in which gender equality is informed about and discussed”; and (6) “Defend gender equality in discussions with peers, colleagues, relatives, and/or friends” (see Reimer et al., 2017). Responses were averaged to create a composite measure of collective action tendencies (α = .92), with high numbers indicating greater intent to engage in collective action toward improving gender balance.

### Results and Discussion

ANCOVAs were conducted to investigate the effect of activating ST paired with feelings of solidarity with women (ST-Similarities) on support for gender balance relative to the neutral control and de-activation (ST-Differences) conditions, statistically controlling for demographic factors.

Focusing first on the *attitudinal support* dependent measure, the omnibus test indicated significant condition differences in attitudinal support for gender balance, *F*(2, 428) = 3.583, *p* = .029, *η_p_*^2^ = .016. Investigating *a priori* pairwise comparisons revealed that women’s self-reported support for gender balance was higher when feelings of solidarity were activated in the context of ST in the ST-Similarities condition (*M* = 6.21, *SE* = .094) relative to the neutral Control condition (*M* = 5.88, *SE* = .085), mean difference = 0.338, *SE* = .127, *p* = .008, 95% CI for Difference [.088, .587], and marginally higher relative to the ST-Differences condition (*M* = 5.99, *SE* = .091), mean difference = 0.221, *SE* = .132, *p* = .094, 95% CI for Difference [−.038, .480]. The mean difference in attitudinal support between the neutral Control and ST-Differences conditions was not significant (*p* = .350). These findings provide robust support for H2 and suggestive evidence of H3: Women managers expressed more support for gender balance when common fate was manipulated in the context of ST compared with a neutral control condition, as well as compared with a condition in which the mediator was “turned off” in the context of ST.

In the same set of analyses for *behavioral intent to support* gender balance (the same two-item composite measure used in Studies 3 through 5), the omnibus test was again significant, *F*(2, 428) = 3.572, *p* = .029, *η_p_*^2^ = .016. *A priori* pairwise comparisons revealed that the ST-Similarities condition increased women’s interest in mentoring and advocating for women (*M* = 3.53, *SE* = .099) relative to the Control condition (*M* = 3.19, *SE* = .090), mean difference = 0.341, *SE* = .134, *p* = .011, 95% CI for Difference [.079, .604], but nonsignificantly relative to the ST-Differences condition (*M* = 3.43, *SE* = .096), mean difference = 0.097, *SE* = .139, *p* = .485, 95% CI for Difference [−.176, .369]. There was a marginally significant difference between the Control and the ST-Differences conditions (mean difference = −0.245, *SE* = .131, *p* = .063, 95% CI for Difference [−.503, .014]). Thus, again providing compelling evidence in support of H2 and some qualified evidence supporting H3, and replicating the focal effect tested in Studies 3 through 5, we find that “turning on” feelings of common fate with other women in the context of personal ST experiences increased women managers’ self-reported intentions to mentor and advocate for women compared with a neutral control condition. However, we failed to cleanly “turn off” the mediator’s effect on women’s intentions to mentor and advocate for women.

Turning to the dependent measure of *policy support* for gender balance, an omnibus test again found significant differences among conditions, *F*(2, 428) = 5.834, *p* = .003, *η_p_*^2^ = .027. Pairwise comparisons demonstrated that women’s policy support was higher in the ST-Similarities condition (*M* = 4.94, *SE* = .139) relative to the neutral Control condition (*M* = 4.30, *SE* = .125), mean difference = 0.635, *SE* = .187, *p* < .001, 95% CI for Difference [.268, 1.002], and nonsignificantly higher relative to the ST-Differences condition (*M* = 4.64, *SE* = .134), mean difference = 0.297, *SE* = .194, *p* = .126, 95% CI for Difference [−.084, .677]. There was a marginally significant difference between the Control and the ST-Differences conditions (mean difference = −0.338, *SE* = .184, *p* = .066, 95% CI for Difference [−.699, .023]). Similar to the two-item measure of behavioral intent to mentor and advocate for women, we find that “turning on” common fate in the context of ST increases policy support compared with a neutral control condition. However, while discouraging the mediator by focusing on differences in the context of ST seems to dampen the effect, it does not completely turn it off.

Finally, we repeated the ANCOVA analysis with the dependent measure of collective action tendencies and again found the omnibus test to be significant, *F*(2, 428) = 3.609, *p* = .028, *η_p_*^2^ = .017. Again, *a priori* pairwise comparisons revealed that women’s collective action tendencies were higher when feelings of solidarity were activated in the context of ST in the ST-Similarities condition (*M* = 5.18, *SE* = .119) relative to the neutral Control condition (*M* = 4.76, *SE* = .108), mean difference = 0.418, *SE* = .160, *p* = .009, 95% CI for Difference [.104, .733], and marginally higher relative to the ST-Differences condition (*M* = 4.86, *SE* = .115), mean difference = 0.320, *SE* = .166, *p* = .055, 95% CI for Difference [−.007, .646]. The mean difference in attitudinal support between the neutral Control and ST-Differences conditions was not significant (*p* = .531). Taken together, these findings provide unambiguous causal support for H2 and qualified support for H3: Women managers expressed greater collective action tendencies when common fate was manipulated in the context of ST compared with a neutral control condition and also a trend toward greater support as compared with when common fate was “turned off” in the context of ST. Regarding the qualified support (found across all focal dependent measures in this study) for the mediation model hypothesized in H3 and empirically supported in Study 5, it is possible that the common fate implicitly activated by manipulating ST was too powerful to “turn off” completely in the ST-Differences condition. Future work might explore ways to more cleanly separate these variables, perhaps via longitudinal designs.

### General Discussion

Our findings complement and develop theory and evidence on ST and women in the workplace, revealing that experiencing systemic ST is a significant driver of women’s support for and actions toward developing gender balance. The main contribution of this research pertains to identifying a novel outcome associated with ST. In addition to ST leading to unambiguously negative performance outcomes and disengagement with respect to the stereotyped domain, we hypothesized and found that ST can lead to increased ingroup support, solidarity, and diversity-relevant change motivation.

Studies 1 and 2 demonstrated that in the male-dominated context of competitive international business and leadership, women who expressed heightened concerns about confirming negative gender stereotypes were more motivated and took more action to ameliorate gender inequity. Studies 3 through 6 demonstrated causally that activating ST increased working women’s support for gender balance in leadership and their intentions to support other women at work via mentoring and sponsoring. Furthermore, women’s stereotype-threat-activated desire to impact change was mediated by their solidarity with other women—feeling that their fight was the collective fight of all women.

Women’s support for gender balance was measured in various ways, including desire to achieve gender balance with respect to numeric representation (Studies 1 and 3–6), providing or intending to provide opportunities to support women through mentoring and sponsoring (Studies 1 and 3–6), addressing women’s experiences of inclusion and belonging (Study 2), and expressing workplace policy support and collective action tendencies (Study 6). Building upon and progressing beyond decades of research showing that ST impedes women’s domain-relevant performance and engagement, we provide initial evidence that women’s support for gender balance can be motivated by ST.

### Implications for Leaders and Organizations

A primary goal of this research was to uncover what factors motivate some—but not all—women, *and especially women leaders*, to use their power, influence, and voice to fight for women’s status and opportunities. Findings suggest that women who recognize and connect with system-wide experiences about being judged based on gender stereotypes are motivated to contribute more to advance gender equity. This has broader implications for managers and leaders striving to develop DEI efforts in their organizations. In addition to designing and implementing interventions to reduce the negative impacts of ST (Kinias & Sim, 2016; Roberson & Kulik, 2007; Schmader & Hall, 2014), organizations should thoughtfully apply interventions that reduce the negative impact of ST while still motivating change to address existing inequities. Work on the “sedative effect” of prejudice reduction strategies has found that alleviating intergroup conflict creates an optimistic illusion of intergroup equality, which diminishes willingness to engage in social change or collective action (Glasford & Dovidio, 2011; Saguy, 2018). Our work suggests that organizations with DEI goals should similarly be cautious about merely reducing *perceptions* of threat and stopping there, before impactful change can be achieved and without eliminating the *sources* of ST (e.g., Brockner & Sherman, 2021; Leslie, 2019).

Organizations might focus on providing resources and support for women to give voice to their own ST experiences without reinforcing gender stereotypes and gender differences (Duguid & Thomas-Hunt, 2015). For women, acknowledging ST experiences may start to break down the bias-perpetuating systems in which they are entrenched (see also Vázquez et al., 2020). Frank conversations and open discussions about ST can be led by senior women who both serve as role models (Cortland & Kinias, 2019) and re-commit to driving gender balance. A note of caution: Although this extra work can be beneficial for the societal structures and organizations women lead and serve, it may come at the nontrivial personal cost of additional unrewarded labor and potential punishment for women (Hekman et al., 2017). However, it can also result in positive downstream professional outcomes: forging close network ties and sharing insider knowledge with other women about how to succeed in male-dominated spaces can lead to acquiring top leadership job placements (Yang et al., 2019).

### Limitations and Future Research

This work is the first to our knowledge to empirically link ST with support for social change, so there are many potential avenues for future research. One particularly fruitful avenue would be to begin to disentangle which facets of ST—for example, concerns about stereotypes that implicate the self versus the group, and that involve judgment coming from the self versus ingroup/outgroup others (Shapiro & Neuberg, 2007)—are more likely to incite various change-relevant behaviors, above and beyond those measured in the current work (e.g., confronting bias; Brands & Rattan, 2020). We also encourage further exploring the details of when—and in what forms—experiences of systemic ST can lead to collective action and ingroup support. For example, such research could investigate whether inducing a performance-dampening experience of acute ST (e.g., a specific difficult leadership task with information about gender differences in performance) would lead women to support gender equity in the same way that our measures and manipulations of more chronic forms of systemic ST did. Relatedly, more work is needed to examine whether the experience of ST itself is enough to elicit ingroup support and collective action, or whether conscious acknowledgment and reflection of these ST experiences is necessary. Some contexts may be too threatening to plausibly open up pathways leading to positive change. Indeed, recent work on women in the military suggests a potential contextual boundary condition: Hypermasculine settings may lead women to distance themselves from other women as a coping response to chronic and severe ST (Veldman et al., 2021). Investigating these questions will be crucial to advancing theory on this topic.

Although our replication of focal effects among women participants varying in age, seniority, education, global location, and industry strongly implies generalizability, we recognize some limits. There are other relevant implications of intersections of identity-based disempowerment with race, ethnicity, and economic deprivation that may influence motivations and behaviors (e.g., Adbi et al., 2021; Bhattacharyya & Berdahl, 2023). Thus, we echo calls for research addressing nonbinary gender identities and intersectionality (Cole, 2009; Ramarajan, 2014; Remedios & Snyder, 2015), and encourage investigations into how experiencing ST relevant to various meaningful social identities (e.g., race, ethnicity, and age) can influence efforts to improve equitable opportunities.

We underscore that although the research reported here focuses on women, scholars should think more broadly about motivating all potential change agents without relying on women and other underrepresented groups to disproportionately lead change efforts. Few articles have done this to date (e.g., Georgeac & Rattan, 2019), and future research efforts might address other leadership-based motivations for gender balance that could be relevant for both male and female business leaders. Men’s relatively advantaged position in society and overrepresentation in leadership roles places them in an influential position for the kind of impactful organizational change management requiring leadership support at the highest levels (Dwivedi et al., 2018; Selvanathan et al., 2020). This highlights the importance of future work addressing the factors involved in predicting men’s support for gender balance.

### Conclusion

Decades of research have documented negative outcomes for women experiencing stereotype threat (ST)—or the fear of being judged through the lens of negative gender stereotypes—with respect to diminished performance, motivation, and engagement in the stereotyped domain. Beyond these effects, we argue and find evidence for a previously unstudied consequence of ST: igniting women’s motivation to support and champion gender balance. Six studies demonstrate that experiences of systemic ST can add fuel to the fire of women’s collective fight for justice, stoking the flames of their ingroup support, solidarity, and collective action. This work underscores the importance of women acknowledging and recognizing their concerns about confirming gender stereotypes, as doing so may be fundamental in motivating their own efforts to foster equity and develop balance. Organizations motivated to achieve gender balance would benefit from thoughtful implementation of workplace interventions designed to reduce objective bias and sources of ST while simultaneously empowering catalysts of change.

## Supplemental Material

sj-docx-1-psp-10.1177_01461672231202630 – Supplemental material for Adding Fuel to the Collective Fire: Stereotype Threat, Solidarity, and Support for Change Supplemental material, sj-docx-1-psp-10.1177_01461672231202630 for Adding Fuel to the Collective Fire: Stereotype Threat, Solidarity, and Support for Change by Clarissa I. Cortland and Zoe Kinias in Personality and Social Psychology Bulletin

## Notes

1. The surveys in Studies 1 and 2 included additional measures irrelevant to the current article’s focal hypotheses, all of which are reported in the SOM.

2. Excluding covariates did not change the pattern of findings or levels of significance. We report all results from analyses including the covariates, as controlling for them provides a more rigorous test of the focal hypotheses (see SOM for all analyses excluding covariates).

3. These data were part of an ongoing climate survey administered at the school, and thus, the question wording and response options were fixed. We acknowledge these limitations and validate them where possible, as well as address them in the studies that follow.

4. Participants also indicated willingness to recommend their school to men considering MBA programs, though responses to this question were not relevant to the focal hypotheses. Analyses with recommendations to men as the outcome demonstrated that ST did not predict women’s recommendations to men, *p* = .226.

5. Excluding covariates does not change the pattern of results for any of the analyses reported in Studies 3 to 6, but the effects of some of the planned contrasts analyses in Study 4 were no longer significant (see SOM for results of all analyses excluding covariates in all studies).

6. The original preregistration included a moderation test of gender identification, which was dropped from the main article due to its exploratory nature and nonsignificant interactions with experimental conditions across all the focal dependent measures.
