# Supplementary material for: Adding Fuel to the Collective Fire: Stereotype Threat, Solidarity, and Support for Change
Source: Pers Soc Psychol Bull. 2023 Oct 21;51(5):808–27. doi: 10.1177/01461672231202630 (PMC11930638; doi:10.1177/01461672231202630)
Supplement: sj-docx-1-psp-10.1177_01461672231202630 – Supplemental material for Adding Fuel to the Collective Fire: Stereotype Threat, Solidarity, and Support for Change [file sj-docx-1-psp-10.1177_01461672231202630.docx]

**SUPPLEMENTAL ONLINE MATERIALS**

**All Analyses Excluding Covariates + Additional Study Information**

The aim of this supplemental document is to report all analyses included in the main text of the paper with covariates excluded, so as to allow for inclusion of these data in any future meta-analyses. We also include any information on study exclusions where applicable, and any additional study information referenced in the main paper, including details on the construct and measurement validation efforts in Studies 1 and 2.

**Study 1**

All living alumni (N=63,229) were invited to participate in the survey, including men (N=51,234) and women (11,967). Responses from male alumni were excluded from the manuscript and from analyses because only the sub-sample of women alumnae were relevant to the tested hypotheses. The social psychological theory behind gender-relevant stereotype threat in professional, male-dominated contexts applied only to the women in the full dataset. Response rate among women alumnae respondents was 11% (response rate was 9.5% for all alumni). The variables relevant to our hypothesis tests were embedded amongst other measures unrelated to gender or stereotype threat. These other measures were part of a larger institutional initiative to understand a wide range of alumni experiences and impact. They included: alumnae contributions to economic development in emerging markets, profit/nonprofit board service, commitment to environmental sustainability, leadership self-perceptions and success narratives, and impacts of the COVID pandemic on alumni’s work experiences.

***Details of external sample collected for validation purposes***. Working women in the U.S. (*N* = 142; median age range: 30-39; 75.4% White, 7% African-American/Black, 4.2% Hispanic/Latinx, 5.6% Asian/Asian-American, 6.3% multiracial, all other = 1.4%) were recruited from Prolific to participate in a paid online study. The results reported here have not been described elsewhere. Participants responded to the focal measures used in Study 1 along with the following additional measures used for validation purposes: (1) the Devaluing sub-scale of the Psychological Disengagement Scale (Major & Schmader, 1998), measuring the extent to which participants agreed it was important or unimportant to do well at work (“I always feel good about myself when I do well at work”; “Being good at my job is an important part of who I am”; “Doing well at work is very important to me”; “I care a great deal about performing well at work”; “It usually doesn’t matter to me one way or another how I do at work,” reverse-scored; all items measured on a scale from 1 = *strongly disagree* to 7 = *strongly agree*; α = .87); (2) the Career-Oriented Commitment scale (Ellemers, de Gilder, & van den Heuvel, 1998), measuring the extent to which participants agreed they felt committed to the goal of advancing at work (“My work is one of the most important things in my life”; “I regularly consider what I could do to get ahead at work”; “The ambitions in my life mainly have to do with my work”; “My work plays a central role in my life”; measured on a scale from 1 = *strongly disagree* to 7 = *strongly agree*; α = .79); and (3) Gender Equity Support (adapted from Bargad & Hyde, 1991), measuring the extent to which participants agreed that society should achieve gender equity (“I want to work to improve women’s status in business and society”; “I care very deeply about men and women having equal opportunities in all respects”; scale from 1 = *strongly disagree* to 7 = *strongly agree*; *r* = .650).

Although our measure of domain engagement focused on work satisfaction, engagement at work can take other forms, including a sense of valuing work and commitment to work. Thus, we conducted a confirmatory factor analysis (CFA) to assess the fit of a model in which our three items included in the main paper loaded onto a latent measure of work satisfaction that correlated with two other validated measures of domain engagement: Major and Schmader’s (1998) Psychological Disengagement scale, and Ellemers et al.’s (1998) Career Oriented Commitment scale. Results revealed that work satisfaction correlated meaningfully with valuing work (estimate = 0.51) and with work commitment (estimate = 0.55), and that this model fit the data relatively well, *χ*^2^_(2)_ = 119.418, *p* < .001, CFI = .931, IFI = .932, TLI = .910, GFI = .879, RMSEA = .098 [.075, .120], *p* = .001. Thus, the work satisfaction measure used in Study 1 taps work engagement similarly to these other two established scales of domain engagement.

Furthermore, to investigate our thinking that the single-item measures of support for gender balance reported in Studies 1 and 2 constitute valid short-form measures of attitudinal and behavioral support, we conducted a second CFA. Here, we created two latent variables. The first was one comprised of an established two-item measure of gender equity support (Bargad & Hyde, 1991) and the two one-item measures of attitudinal support from Studies 1 and 2, for a total of four items loading onto one latent variable measuring attitudinal support. The second latent variable was comprised of the two-item measure of behavioral support in Study 1 and the one-item measure of behavioral support in Study 2, for a total of three items loading onto a second latent variable measuring behavioral support of gender balance. Results revealed that a model comprised of these two latent variables (attitudinal and behavioral support for gender balance) set to be correlated with each other (estimate = 0.56) fit the data well, *χ*^2^_(2)_ = 57.688, *p* < .001, CFI = .927, IFI = .928, TLI = .882, GFI = .895, RMSEA = .156 [.116, .198], *p* < .001.

Importantly, each of the (four) items tapping into attitudinal support for gender balance loaded significantly onto the relevant latent variable (*β* ≥ 0.62, *p* < .001), and the standardized loading of the single item from Study 1 with the overall latent measure of attitudinal support was *β* = 0.87, *p* < .001. The standardized loading of the single item from Study 2 with the overall latent measure of attitudinal support was *β* = 0.82, *p* < .001. Finally, the three items tapping into behavioral support for gender balance all loaded significantly onto the relevant latent variable (*β* ≥ 0.82, *p* < .001), and the standardized loading of the single item from Study 2 with the overall latent measure of behavioral support was *β* = 0.82, *p* < .001, indicating the single-item measures used in Studies 1 and 2 constitute valid short-form measures of attitudinal and behavioral support.

***Analyses excluding covariates***. See Supplemental Table 1 for the results of the linear regressions testing our main hypotheses. We first regressed the composite measure of workplace satisfaction on women’s stereotype threat concerns. Stereotype threat concerns significantly and negatively predicted women’s reported workplace satisfaction, *b* = -0.052, *SE* = 0.022, *t*(1353) = -2.381, *p* = .017, 95% CI [-0.096, -0.009]. Next, we regressed women’s interest in seeing efforts to increase the representation of women in leadership on stereotype threat concerns. Stereotype threat concerns significantly and positively predicted attitudinal support of gender balance, *b* = 0.210, *SE* = 0.024, *t*(1361) = 8.725, *p* < .001, 95% CI [0.163, 0.258]. Finally, we regressed women’s self-reported behavioral support of gender balance on stereotype threat and found that women’s increased concerns about confirming gender stereotypes in their careers significantly and positively predicted their contributions to efforts developing gender balance by mentoring and advocating for other women, *b* = 0.123, *SE* = 0.021, *t*(1113) = 5.718, *p* < .001, 95% CI [0.081, 0.165].

**Study 2**

All full-time enrolled MBA students (N_population_ = 3,115; N_female_ = 1,059; M_age_ = 29) were invited to participate in the survey, including men and women. Just as in Study 1, responses from male MBA students were excluded from the manuscript and from analyses because only the sub-sample of female MBA students were relevant to the tested hypotheses. The variables relevant to our hypothesis tests were embedded amongst other question items, including perceptions of gender bias in specific school spaces or contexts, perceptions of voice and speaking up about gender balance, awareness of various school efforts to improve the gender climate, perceptions of different leadership identities, short and long-term career goals, and other various open-ended questions about students’ perceptions of the gender climate at their school.

***Details of external sample collected for validation purposes***. Participants (*N* = 142; see Study 1 above for demographics of this sample) responded to the focal measures used in Study 2 along with the following additional measures used for validation purposes: (1) Perceived Gender Bias (adapted from Thomas et al., 2020), measuring the extent to which participants perceived bias against their gender ingroup (“Do you think people from your gender group are discriminated against at your work?”; “Do you think people from your gender group are treated unfairly at your work?”; “To what extent do you think your gender group is disadvantaged at your work?”; scale from 1 = *not at all* to 7 = *very much*; α = .93); (2) Experienced Gender Bias (adapted from Thomas et al., 2020), measuring the extent to which participants experienced bias against their gender ingroup (“Have you personally been discriminated against due to your gender at work?”; “Have you personally been treated unfairly due to your gender at work?”; “To what extent are you disadvantaged due to your gender at work?”; scale from 1 = *not at all* to 7 = *very much*; α = .94); (3) the Devaluing sub-scale of the Psychological Disengagement Scale (Major & Schmader, 1998; see details described above in Study 1); (4) the Career-Oriented Commitment scale (Ellemers, de Gilder, & van den Heuvel, 1998; see details described above in Study 1); and (5) Gender Equity Support (adapted from Bargad & Hyde, 1991; see details described above in Study 1).

First, to investigate our thinking that the single-item measures of perceived and experienced gender bias reported in Study 2 constitute valid short-form measures of perceiving and experiencing gender bias, we conducted two CFAs. These included the three-item measures of perceived gender bias and experienced gender bias (Thomas et al., 2020) and our one-item measures loaded onto their relevant latent construct. The CFA model for the latent construct of perceived gender bias fit the data well, *χ*^2^_(2)_ = 0.951, *p* = .622, CFI = 1.000, IFI = 1.002, TLI = 1.006, GFI = .997, RMSEA < .001 [.000, .134], *p* = .713. Importantly, each of the (four) items loaded significantly onto the latent variable (*β* ≥ 0.64, *p* < .001, and the standardized loading of the target item used in Study 2 with the overall latent measurement of perceived gender bias was *β* = 0.64, *p* < .001. Focusing next on experienced gender bias, results revealed that a model comprised of the latent variable of experienced gender bias fit the data well, *χ*^2^_(2)_ = 2.033, *p* = .362, CFI = 1.000, IFI = 1.000, TLI = 1.000, GFI = .993, RMSEA = .011 [.000, .167], *p* = .481. Notably, each of the (four) items loaded significantly onto the latent variable (*β* ≥ 0.68, *p* < .001, and the standardized loading of the target item with the overall latent measurement of experienced gender bias was *β* = 0.68, *p* < .001.

Furthermore, although the measure of domain engagement used in Study 2 focused on school commitment, engagement can take other forms, as noted above. Thus, we conducted a CFA to assess the extent to which our items measuring commitment loaded onto a latent construct of domain engagement that correlated with the same two established measures of domain engagement tested in the CFA in Study 1. Results revealed that measure of commitment used in Study 2 correlated meaningfully with Major & Schmader’s (1998) valuing work scale (estimate = 0.69) and with Ellemers et al.’s (1998) career-oriented commitment scale (estimate = 0.68), and that this model fit the data relatively well, *χ*^2^_(2)_ = 164.520, *p* < .001, CFI = .923, IFI = .924, TLI = .906, GFI = .856, RMSEA = .093 [.074, .112], *p* < .001. Thus, the commitment measure used in Study 2 taps domain engagement similarly to these other two established forms of domain engagement.

See results of CFA reported in Study 1, above, for findings regarding the validation of the single-item measures of support for gender balance used in Study 2. All analyses suggest the target items used in Study 2 tap into the applicable underlying latent variables and constitute valid short-form measures of their applicable constructs.

***Analyses excluding covariates***. See Supplemental Table 2 for the results of the linear regression analyses testing our main hypotheses. We first regressed our composite measure of school belonging on women’s concerns about confirming negative gender stereotypes at their school (mean-centered), and found that stereotype threat concerns significantly and negatively predict women’s school belonging, *b* = -0.123, *SE* = 0.034, *t*(396) = -3.602, *p* < .001, 95% CI [-0.192, -0.056]. Regressing women’s willingness to recommend their school to prospective MBA students on women’s reported stereotype threat concerns revealed that stereotype threat was a significant predictor, *b* = -0.195, *SE* = 0.040, *t*(396) = -4.856, *p* < .001, 95% CI [-0.275, -0.116]. Next, we regressed women MBA student’s desire to see efforts to correct the gender imbalance at their school on stereotype threat concerns, and found that stereotype threat was significantly and positively associated with women’s interest in seeing positive change happen with respect to the gender climate at their school, *b* = 0.192, *SE* = 0.027, *t*(392) = 7.109, *p* < .001, 95% CI [0.139, 0.245]. Finally, we regressed women’s self-reported behavioral contribution to improving the gender climate at their school on stereotype threat concerns, and found that stereotype threat was a significant predictor, *b* = 0.258, *SE* = 0.063, *t*(211) = 4.086, *p* < .001, 95% CI [0.133, 0.382].

**Study 3**

Participants had varied levels of education (39% of sample reported less than bachelor’s level) and job status (27% of sample reported working only part-time, and 47% of sample reported having no management experience). Participants were recruited from two online participant recruitment systems: Amazon Mechanical Turk (MTurk; N = 237) and Prolific Academic (N = 290). The two datasets were merged and tested for moderation, which was not present (all *p*’s for interaction of sample X condition > 0.5). Therefore, results are reported for analyses conducted on the full merged sample with an indicator variable for online sample included as a covariate.

Participants in the *stereotype threat condition* were given the following instructions:

“Please think back to a time while you were working when you were being evaluated or were engaging in an action where women are negatively stereotyped. Please think about a time when you were concerned that your actions, performance, or behaviors could be used to gauge your personal abilities and that others might judge you based on your actions and these negative stereotypes. For example, this might have been during a performance review at work, speaking up in a staff meeting at work when you had an idea to share, negotiating for things like pay or benefits, or applying for a promotion at work. Think about how you felt, both physically and emotionally, in that moment.”

***Analyses excluding covariates***. Analyses of variance (ANOVAs) were conducted to investigate mean differences between conditions, without statistically controlling for age, education, racial/ethnic minority status, political orientation, and online sample. Women’s attitudinal support for improving gender balance was examined as a function of whether stereotype threat – concerns about confirming negative gender stereotypes – was salient or not. Women’s desire to see efforts to increase the representation of women in leadership positions was higher when women wrote about a stereotype threat experience at work compared with when they wrote about what they did last Tuesday in the control condition, *F*(1, 507) = 5.431, *p* = .020, *η_p_*^2^ = .011. Furthermore, women’s interest in investing their own personal time and effort in mentoring and advocating for women was also higher in the stereotype threat condition versus the control condition, *F*(1, 515) = 6.698, *p* = .010, *η_p_*^2^ = .013.

**Study 4**

Participants in the negative affect condition were given the following instructions:

“Please think back to a time when you failed to reach a goal at work. For example, this might have been related to your performance, company revenue/sales, meeting a deadline, deliverables, etc. Think about how you felt, both physically and emotionally, in that moment.”

In addition to the measures reported in the manuscript, an additional measure of self-reported policy support was included in this study. Participants indicated their level of opposition or support to the following five policy proposals on a scale ranging from 1 = *strongly oppose* to 7 = *strongly support*: (1) “A policy whereby a woman can get hired over a male applicant as long as the woman meets a minimal level of qualifications. Under this policy, it is possible for a woman to get hired even if she is relatively less qualified than a male applicant.” (2) “A gender-blind policy whereby a candidate's gender is completely ignored throughout the entire employment procedure (i.e., in both the recruiting and hiring stage).” (3) “A "tie-breaker" policy in which a woman is selected over a male applicant when the two applicants are equally qualified.” (4) “A policy through which women can receive supplemental training to prepare them for the selection process. However, gender is not considered at the hiring stage.” (5) “A policy that requires the company to make extra efforts to get women to apply for job openings but does not take gender into consideration at the hiring stage. Examples of such extra efforts include advertising in magazines with a high female readership or recruiting applicants from women's groups.” There were no consistent differences among and between the condition means for policy support – analyzed as either a composite measure (α = .50) or looking at each of the five items individually (all *p*s > .05).

ANCOVAs were conducted to investigate the effect of stereotype threat on the composite measure of *policy support* for gender balance relative to the neutral control and negative work affect conditions, statistically controlling for demographic factors. The omnibus test was non-significant, *F*(2, 568) = 1.887, *p* = .152, *η_p_*^2^ = .007. Planned contrasts analyses revealed that women’s policy support was not statistically different when comparing the stereotype threat condition (*M* = 4.82, *SE* = .067) to the control condition (*M* = 4.73, *SE* = .059), *F*(1, 568) = .972, *p* = .325, *η_p_*^2^ = .002, nor compared to the negative affect condition (*M* = 4.64, *SE* = .062), though this latter difference was marginally significant, *F*(1, 568) = 3.764, *p* = .053, *η_p_*^2^ = .007. There was no difference between the control and the negative affect conditions (*p* = .294), and no difference in policy support between the stereotype threat condition and the average of the other two conditions combined, *F*(1, 568) = 2.785, *p* = .096, *η_p_*^2^ = .005.

***Analyses excluding covariates***. Analyses of variance (ANOVAs) were conducted to investigate mean differences between conditions, without statistically controlling for age, education, racial/ethnic minority status, and political orientation. The omnibus test of differences between the three condition means on attitudinal support for gender balance was not significant, *F*(2, 573) = 2.036, *p* = .132, *η_p_*^2^ = .007. Testing the first planned contrast, results revealed that women’s desire to see efforts to increase the representation of women in leadership positions was marginally higher when they wrote about a stereotype threat experience at work compared with when they wrote about what they did last Tuesday in the control condition, *F*(1, 573) = 3.619, *p* = .058, *η_p_*^2^ = .006. Testing the second planned contrast, women were more interested in seeing efforts to advance gender balance in leadership after writing about a work-related stereotype threat experience compared with when they wrote about a more general negative work experience not related to stereotype threat, *F*(1, 573) = 2.605, *p* = .107, *η_p_*^2^ = .005. There was no difference between the Tuesday control and negative affect conditions, *F*(1, 573) = 0.067, *p* = 0.796,*η_p_*^2^ = .000. Finally, women who reflected on a personal experience of stereotype threat at work expressed significantly more support for gender balance in leadership than the average of the other two conditions, *F*(1, 573) = 3.970, *p* = .047, *η_p_*^2^ = .007.

We conducted the same set of analyses for the measure of policy support for gender balance. The omnibus test of differences between the three condition means on attitudinal support for gender balance was not significant, *F*(2, 573) = 1.029, *p* = .358, *η_p_*^2^ = .004. Results from the first planned contrast reveal that women’s expressed support for organizational policies supporting gender balance was no different in the stereotype threat condition compared to the Tuesday control condition, *F*(1, 573) = 1.049, *p* = .306, *η_p_*^2^ = .002. Policy support was no different in the stereotype threat condition compared to the negative affect condition, *F*(1, 573) = 1.971, *p* = .161, *η_p_*^2^ = .003. There was no significant difference between the Tuesday control and the negative affect conditions, *F*(1, 573) = .188, *p* = .664, *η_p_*^2^ = .000. Finally, there was no difference in policy support when comparing the stereotype threat condition to the average of the other two conditions combined, *F*(1, 573) = 1.904, *p* = .168, *η_p_*^2^ = .003.

We conducted the same set of analyses for the measure of behavioral intent to support gender balance. The omnibus test of differences between the three condition means on attitudinal support for gender balance was significant, *F*(2, 573) = 3.035, *p* = .049, *η_p_*^2^ = .010. Results from the first planned contrast reveal that women’s interest in investing their own personal time and effort to mentoring and advocating for women was higher in the stereotype threat condition compared to the Tuesday control condition, *F*(1, 573) = 6.068, *p* = .014, *η_p_*^2^ = .010. Interest in advancing gender balance via intentions to mentor and advocate for women was higher in the stereotype threat condition than the negative affect condition, though this difference was only trending and did not reach conventional levels of significance, *F*(1, 573) = 1.949, *p* = .163, *η_p_*^2^ = .003. There was no significant difference between the Tuesday control and the negative affect conditions, *F*(1, 573) = 1.164, *p* = .281, *η_p_*^2^ = .002. Finally, we found the predicted effect that stereotype threat led to greater intentions to support other women than the average of the other two conditions combined, *F*(1, 573) = 4.759, *p* = .030, *η_p_*^2^ = .008.

Finally, participants indicated how interested they would be in investing their own time and effort in mentoring and advocating for men and/or boys in their industry/profession. The omnibus tests and all four planned contrasts revealed no significant mean differences between the conditions (all *p*s > .4).

**Study 5**

***Analyses excluding covariates***. Analyses of variance (ANOVAs) were conducted to investigate mean differences between conditions, without statistically controlling for age, education, racial/ethnic minority status, and political orientation. Women’s perceived common fate with other women was higher when women wrote about a stereotype threat experience at work compared with when they wrote about what they did last Tuesday in the control condition, *F*(1, 380) = 5.546, *p* = .019, *η_p_*^2^ = .014. Furthermore, women’s interest in seeing efforts to correct the gender imbalance in high-impact leadership positions is higher in the stereotype threat condition compared to the control condition, *F*(1, 380) = 5.859, *p* = .016, *η_p_*^2^ = .015. Women’s support for gender equality as measured via the additional two-item composite measure was also higher in the stereotype threat condition versus the control condition, *F*(1, 380) = 11.049, *p* = .001, *η_p_*^2^ = .028. Finally, women reported increased interest in investing their own personal time and effort to mentoring and advocating for women in the stereotype threat condition as compared to the control condition, *F*(1, 380) = 9.419, *p* = .002, *η_p_*^2^ = .024.

To test for the significance of the indirect pathway from stereotype threat to support for gender balance through the mediator of perceived common fate with other women, we used the PROCESS macro by Andrew Hayes (Model 4; Hayes, 2012). Three models were specified to test the mediating process on each of the 3 outcomes: (1) interest in seeing efforts to increase female representation in leadership positions; (2) attitudinal support for gender equality; and (3) behavioral intentions to mentor and advocate for women. Each PROCESS command was run with bootstrapping specified at 10,000 samples, and excluding the covariates reported in the main article. As shown in Supplemental Table 3, perceived common fate with other women significantly mediated the relationship between stereotype threat and support for gender balance – analyzed via two measures of attitudinal support and a third measure of behavioral intentions. (See Supplemental Table 3 for path coefficients and confidence intervals of all mediation models.)

**Study 6**

***Analyses excluding covariates***. Analyses of variance (ANOVAs) were conducted to investigate mean differences between conditions, without statistically controlling for age, education, racial/ethnic minority status, and political orientation. Focusing first on the *attitudinal support* dependent measure, the omnibus test indicated significant condition differences in attitudinal support for gender balance, *F*(2, 432) = 4.753, *p* = .031, *η_p_*^2^ = .016. Investigating *a priori* pairwise comparisons revealed that women’s self-reported support for gender balance was higher when feelings of solidarity were activated in the context of stereotype threat in the ST-Similarities condition (*M* = 6.23, *SE* = .102) relative to the neutral control condition (*M* = 5.87, *SE* = .092), Mean Difference = 0.360, *SE* = .137, *p* = .009, 95% CI for Difference [.090, .630], and marginally higher relative to the ST-Differences condition (*M* = 5.99, *SE* = .098), Mean Difference = 0.236, *SE* = .141, *p* = .095, 95% CI for Difference [-.041, .513]. The mean difference in attitudinal support between the neutral control and ST-Differences conditions was not significant (*p* = .357).

In the same set of analyses for *behavioral intent to support* gender balance, the omnibus test was significant, *F*(2, 432) = 3.656, *p* = .027, *η_p_*^2^ = .017. *A priori* pairwise comparisons revealed that the ST-Similarities condition increased women’s interest in mentoring and advocating for women (*M* = 3.52, *SE* = .102) relative to the control condition (*M* = 3.18, *SE* = .093), Mean Difference = 0.344, *SE* = .138, *p* = .013, 95% CI for Difference [.073, .615], but not relative to the ST-Differences condition (*M* = 3.46, *SE* = .098), Mean Difference = 0.064, *SE* = .141, *p* = .649, 95% CI for Difference [-.213, .342]. There was a significant difference between the control and the ST-Differences conditions (Mean Difference = -0.280, *SE* = .135, *p* = .039, 95% CI for Difference [-.544, -.015]).

Turning to the dependent measure of *policy support* for gender balance, an omnibus test found significant differences among conditions, *F*(2, 432) = 5.776, *p* = .003, *η_p_*^2^ = .026. Pairwise comparisons demonstrated that women’s policy support was higher in the ST-Similarities condition (*M* = 4.96, *SE* = .142) relative to the neutral control condition (*M* = 4.30, *SE* = .129), Mean Difference = 0.652, *SE* = .192, *p* < .001, 95% CI for Difference [.274, 1.029], but not significantly higher relative to the ST-Differences condition (*M* = 4.62, *SE* = .136), Mean Difference = 0.333, *SE* = .197, *p* = .091, 95% CI for Difference [-.054, .721]. There was a marginal difference between the control and the ST-Differences conditions (Mean Difference = -0.318, *SE* = .188, *p* = .091, 95% CI for Difference [-.687, .051]).

Finally, we repeated the above ANCOVA analysis with the dependent measure of collective action tendencies, and found the omnibus test to be significant, *F*(2, 432) = 3.117, *p* = .045, *η_p_*^2^ = .014. Again, *a priori* pairwise comparisons revealed that women’s collective action tendencies were higher when feelings of solidarity were activated in the context of stereotype threat in the ST-Similarities condition (*M* = 5.19, *SE* = .139) relative to the neutral control condition (*M* = 4.73, *SE* = .126), Mean Difference = 0.461, *SE* = .187, *p* = .014, 95% CI for Difference [.093, .829], and marginally higher relative to the ST-Differences condition (*M* = 4.87, *SE* = .133), Mean Difference = 0.321, *SE* = .192, *p* = .095, 95% CI for Difference [-.057, .700]. The mean difference in attitudinal support between the neutral control and ST-Differences conditions was not significant (*p* = .447).

Supplemental Table 1

*Study 1: Estimates from Series of Linear Regressions, Excluding Covariates*

| Predictors: |  | Work Satisfaction | | |  | Attitudinal Support | | |  | Behavioral Support | | |
| --- | --- | --- | --- | --- | --- | --- | --- | --- | --- | --- | --- | --- |
|  |  | *β* | *B* | *SE* |  | *β* | *B* | *SE* |  | *β* | *B* | *SE* |
| Intercept |  | — | 3.57** | .03 |  | — | 3.83** | .03 |  | — | 3.41** | .03 |
| Stereotype Threat |  | -.06* | -.05* | .02 |  | .23*** | .21*** | .02 |  | .17*** | .12*** | .02 |
|  |  |  |  |  |  |  |  |  |  |  |  |  |
| *R*^2^ |  | .00 | | |  | .05 | | |  | .03 | | |
| *F* for change in *R*^2^ |  | 5.67* | | |  | 76.12*** | | |  | 32.69*** | | |

*Note.* The stereotype threat predictor was centered according to the overall sample mean. The *β* estimates refer to the standardized regression coefficients. The *B* estimates refer to unstandardized regression coefficients. *SE* = standard error. *N* = 1,286. * *p* < .05, ** *p* < .01, *** *p* < .001.

Supplemental Table 2

*Study 2: Estimates from Series of Linear Regressions, Excluding Covariates*

| Predictors: |  | School Belonging | | |  | Recommend to Women | | |  | Attitudinal Support | | |  | Behavioral Support | | |
| --- | --- | --- | --- | --- | --- | --- | --- | --- | --- | --- | --- | --- | --- | --- | --- | --- |
|  |  | *β* | *B* | *SE* |  | *β* | *B* | *SE* |  | *β* | *B* | *SE* |  | *β* | *B* | *SE* |
| Intercept |  | — | 4.02*** | .05 |  | — | 4.48*** | .05 |  | — | 2.45*** | .04 |  | — | 2.69*** | .09 |
| Stereotype Threat |  | -.18*** | -.12*** | .03 |  | -.24*** | -.20*** | .04 |  | .34*** | .19*** | .03 |  | .27*** | .26*** | .06 |
|  |  |  |  |  |  |  |  |  |  |  |  |  |  |  |  |  |
| *R*^2^ |  | .03 | | |  | .06 | | |  | .11 | | |  | .07 | | |
| *F* for change in *R*^2^ |  | 12.98*** | | |  | 23.58*** | | |  | 50.54*** | | |  | 16.70*** | | |

*Note.* The stereotype threat predictor was centered according to the overall sample mean. The *β* estimates refer to the standardized regression coefficients. The *B* estimates refer to unstandardized regression coefficients. *SE* = standard error. *N* = 398. * *p* < .05, ** *p* < .01, *** *p* < .001.

Supplemental Table 3

*Study 5: Path Coefficients and Confidence Intervals of Mediational Models, Excluding Covariates (N=380)*

|  | Attitudinal Support for Gender Balance (single item) | Attitudinal Support for Gender Balance (two-item composite) | Behavioral Intent to Support Gender Balance |
| --- | --- | --- | --- |
| *a* | .294* (.125) | .294* (.125) | .294* (.125) |
| *b* | .150*** (.022) | .446*** (.033) | .495*** (.043) |
| *c* | .138* (.057) | .327** (.099) | .371** (.121) |
| *c'* | .094 (.055) | .196* (.082) | .225* (.105) |
| 95% CI of the indirect effect | [.009, .092] | [.026, .248] | [.029, .279] |

*Notes: a* denotes the path of the effect of stereotype threat experience salience on perceived common fate with women. *b* denotes the path of the mediator’s (perceived common fate) effect on the dependent variable. *c*' denotes the direct effect of stereotype threat experience salience on the dependent variable. *c* denotes the total effect of stereotype threat experience salience on the dependent variable. Standard errors are in parentheses. **p*<.05, ***p*<.01, ****p*<.001

Supplemental Table 4

*Studies 3-6: Summary of Cell Means and Distribution of Exclusions*

|  | Condition | *n* | Excluded Cases |
| --- | --- | --- | --- |
| **Study 3**  (N=506) | Stereotype Threat | 235 | 15 |
|  | Control | 271 | 19 |
| **Study 4**  (N=576) | Stereotype Threat | 165 | 30 |
|  | Control | 217 | 28 |
|  | Negative Affect | 194 | 30 |
| **Study 5**  (N=380) | Stereotype Threat | 198 | 51 |
|  | Control | 182 | 18 |
| **Study 6**  (N=435) | Stereotype Threat-Similarities | 132 | 21 |
|  | Control | 160 | 19 |
|  | Stereotype Threat-Differences | 143 | 19 |
